# Large Impact of Eurasian Lynx Predation on Roe Deer Population Dynamics

**DOI:** 10.1371/journal.pone.0120570

**Published:** 2015-03-25

**Authors:** Henrik Andrén, Olof Liberg

**Affiliations:** Grimsö Wildlife Research Station, Department of Ecology, Swedish University of Agricultural Sciences, Riddarhyttan, Sweden; University of Queensland, AUSTRALIA

## Abstract

The effects of predation on ungulate populations depend on several factors. One of the most important factors is the proportion of predation that is additive or compensatory respectively to other mortality in the prey, i.e., the relative effect of top-down and bottom-up processes. We estimated Eurasian lynx (*Lynx lynx*) kill rate on roe deer (*Capreolus capreolus*) using radio-collared lynx. Kill rate was strongly affected by lynx social status. For males it was 4.85 ± 1.30 S.E. roe deer per 30 days, for females with kittens 6.23 ± 0.83 S.E. and for solitary females 2.71 ± 0.47 S.E. We found very weak support for effects of prey density (both for Type I (linear) and Type II (non-linear) functional responses) and of season (winter, summer) on lynx kill rate. Additionally, we analysed the growth rate in a roe deer population from 1985 to 2005 in an area, which lynx naturally re-colonized in 1996. The annual roe deer growth rate was lower after lynx re-colonized the study area, but it was also negatively influenced by roe deer density. Before lynx colonized the area roe deer growth rate was λ = 1.079 (± 0.061 S.E.), while after lynx re-colonization it was λ = 0.94 (± 0.051 S.E.). Thus, the growth rate in the roe deer population decreased by Δλ = 0.14 (± 0.080 S.E.) after lynx re-colonized the study area, which corresponded to the estimated lynx predation rate on roe deer (0.11 ± 0.042 S.E.), suggesting that lynx predation was mainly additive to other mortality in roe deer. To conclude, this study suggests that lynx predation together with density dependent factors both influence the roe deer population dynamics. Thus, both top-down and bottom-up processes operated at the same time in this predator-prey system.

## Introduction

The effects of predation on ungulate populations depend on several factors [1]. One of the most important factors is the proportion of predation that is additive versus compensatory to other mortality in the prey, i.e., the relative effect of top-down and bottom-up processes. If most or all of the predation is only compensatory to other mortality, then predation will have little or no impact on the prey population. In that case, the predator takes only a “doomed surplus” of the population [2, 3, 4] and the prey population is mainly regulated from bottom-up. However, top-down and bottom-up processes can operate simultaneously, i.e. prey population growth rate is density dependent and predation is additive. In the absence of large predators, ungulate population dynamics are often determined by a combination of density dependent factors and variation in the environment [5]. Natural re-colonization or introduction of predators into systems where they have been absent for a long period can be used to evaluate the importance of predation, but it is important to control for other factors that might cause decline in prey abundance [6]. Vucetich et al. [7] found that the decline in elk (*Cervus canadensis*) population in Yellowstone national park after the introduction of wolves (*Canis lupus*) in 1996, could be explained by increased human elk harvest and weather conditions (multi-year drought that reduced the reproduction) rather than by wolf predation. Sometimes predation is only the proximate cause of death in ungulates with other factors, like starvation, are the ultimate cause [8, 9, 10].

Eurasian lynx (*Lynx lynx*) (hereafter called “lynx”) prey upon several different prey species, but roe deer (*Capreolus capreolus*) is the preferred prey when available [11, 12, 13, 14, 15]. Jedrzejewska et al. [16] assessed the role of lynx in roe deer population dynamics, by analysing the effect of human control of lynx in eastern Poland during the 20^th^ century. The roe deer population irrupted in the absence of lynx and it declined as lynx re-colonized the area, suggesting that lynx predation was additive to other mortality in roe deer. Molinari-Jobin et al. [17] found lynx predation accounted for 24–37% of all known roe deer mortalities in Switzerland. A Europe wide comparison of roe deer densities indicated that both plant productivity and the presence of large predators (lynx and/or wolf) affected roe deer density [18], and the effect of predation was stronger in environments with lower plant production. Nilsen et al. [19] found a Type II functional response [20] for lynx with a very rapid increase in kill rate at low roe deer density, a typical pattern for a highly specialised predator like the lynx [21, 22]. The annual survival of prime age roe deer decreased from 0.79 to 0.61, after lynx recolonized an area in the Bavarian forest [23]. Similarly, Melis et al. [24] reported a much higher total mortality in roe deer, especially for adult females, in areas with both hunting and lynx predation. Altogether these findings suggest that lynx predation on roe deer is largely additive [24].

The aim of this paper was to estimate the impact of lynx predation on a roe deer population in south-central Sweden. We estimated lynx kill rate and factors affecting this, i.e., lynx social status (females with kittens, males and solitary females), season and roe deer density (Type I and Type II functional responses), in an area with known lynx and roe deer densities. Based on this, we could calculate the predation rate, i.e., the proportion of the roe deer population killed by the lynx population per time unit. Furthermore, we examined the change in population growth rate of roe deer from the period before (1985–1995) to the period after (1996–2005) when lynx naturally re-colonized the area, to test whether lynx predation was mainly additive or compensatory to other roe deer mortality. We controlled for the effect of roe deer harvest, roe deer density [25], snow depth, because it can influence roe deer winter survival [26], vole numbers (*Microtus* and *Chletrionomys* spp) and red fox (*Vulpes vulpes*) numbers, because early fawn survival has been shown to be affected by red fox numbers at low vole densities [27, 28].

## Material and Methods

### Study area

**Fig 1:**
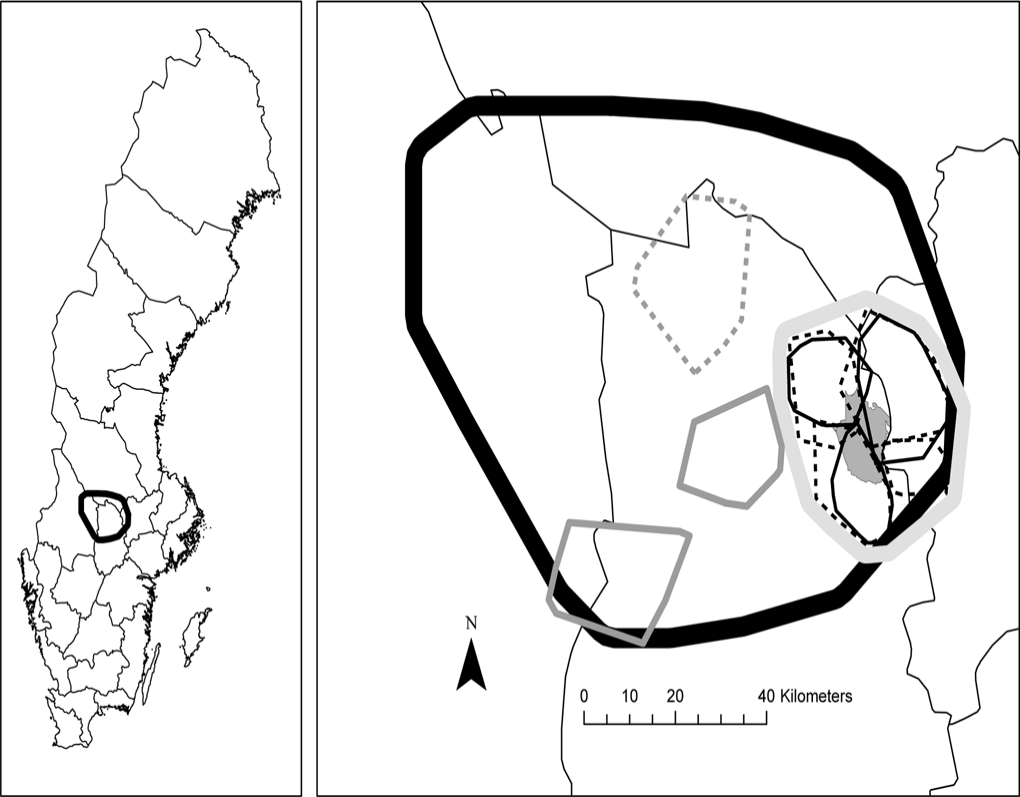
The study areas at three nested spatial scales. Lynx were captured and roe deer surveys were performed at the largest spatial scale (8000 km^2^, thick black line). The number of lynx and roe deer were known within the medium-sized area (2000 km^2^, thick light grey line). Long-term wildlife surveys were performed within Grimsö wildlife research area (130 km^2^, dark grey polygon). The three female (thin black line) and four male (thin black dotted line) lynx home ranges within sub-area in winter 1998/1999, as well as some other examples of female (thin dark grey line) and male (thin dark grey dotted line) lynx home ranges within the largest study area. County borders are also given on both maps.

Our study was made at three different spatial scales with smaller study areas nested within larger. The largest study area was 8000 km^2^ and covering parts of the counties Örebro, Värmland, Dalarna and Västmanland in south-central Sweden (59°15'N - 60°15'N, 13°30'E - ;15°45'E, Fig. 1). We had data on roe deer density for this entire area, and it also contained the home ranges of all the radio-marked lynx included in the study. Within this largest area we had a medium sized area (1200 km^2^), from which we had data on both numbers of lynx and roe deer. Finally, at the smallest scale we used Grimsö wildlife research area (130 km^2^, 59°36'N—59°46'N, 15°20'E—15°32'E) nested within the former area. From this area we have long-term wildlife survey data for a number of different species [for details see 27].

The largest area is on the border between the boreal and boreo-nemoral zone [29]. Norway spruce (*Picea abies*) and Scots pine (*Pinus sylvestris*) dominate the forest, but birch (*Betula pubescens* and *B*. *verrucosa*) and aspen (*Populus tremula*) are mixed into the forest, especially in the young age classes. The forest is managed for pulp and timber, which creates a forest mosaic of even-aged forest stands. Agricultural land covers about 5% of the landscape, with higher proportion in the south. Mean human density is approximately 17 individuals/km^2^, and also increases towards the south. Roe deer is the main prey for lynx in the study area, with mountain hare (*Lepus timidus*), black grouse (*Tetrao tetrix*) and capercaillie (*T*. *urogallus*) as alternative prey [11, 30]. Roe deer is hunted throughout the study area (on all three spatial scales).

In the early 20th century, abundance of lynx was very low and the distribution was restricted in Sweden [31]. Lynx was completely protected in Sweden between 1927 and 1942 and lynx numbers increased. After a temporary decline in the 1980´s, lynx growth and expansion accelerated in the 1990´s especially in south-central Sweden, as a response to a strong increase of the roe deer population during late 1980’s and early 1990’s [32]. Lynx naturally re-colonized Grimsö wildlife research area in 1995–1996 and the first known reproduction occurred in summer of 1996 [33]. After being absent for more than 30 years, the density of lynx went from virtual absence to about 1 lynx per 100 km^2^ in the area around Grimsö within one year [33].

### Ethics statement

This study is based on radio-collared lynx. A variety of methods were used to live-capture and radio-mark lynx; walk-through box-traps, spring-loaded wire foot-snares and trained hounds that forced the lynx at bay, on the ground or up in a tree. The box-traps were constructed in wood and sometimes baited with lynx urine. They were placed on known lynx trails and checked daily from November to April. The foot-snares were placed at fresh lynx killed prey found by chance, by snow tracking, or by monitoring radio-collared prey. Springs were attached to the snares´ anchor wire to dampen the force of escape attempts. The snares were continually monitored using radio-alarms attached to them. The reaction time from capture till handling was less than 15 minutes. The trained dogs were released on a fresh lynx trail after personnel had roughly located the lynx through snow tracking. Usually only one dog was used at a time. The dog chased the lynx until it climbed a tree or sought refuge on the ground or under rocks, and kept it there at bay until the catching personnel arrived. Lynx were immobilised with a mixture of ketamine (5 mg/kg) and medetomidine (0.2 mg/kg) [34]. All drugs were injected intra-muscularly by darting with a blowpipe or gas-powered capture gun. Lynx were fitted with VHF radio-collars (Telonics MOD335 or MOD400NH, Telonics Inc., Mesa, AZ, USA). The handling protocol [35] for lynx has been examined by the Swedish Animal Ethics Committee and fulfils their ethical requirements for research on wild animals (permits C275/95 and C16/0). Lynx is a protected species in the EU Habitat Directive (Annex II and IV). The Swedish Environmental Protection Agency issued permit to perform research on lynx and to capture and immobilize lynx on both state-owned and private land within the entire study area (permit Dnr 410–5531–98 Nf). Special permits for our other research activities (e.g. radio-tracking, searching for killed roe deer, roe deer survey) were not required, neither on state-owned nor on private land.

### Estimating kill rate

We estimated lynx kill rates (number of roe deer killed per 30 days) during limited periods (10–30 days) with intensive day and night monitoring of radio-marked lynx from June 1996 to December 1999. The data were split into two different seasons, winter (1 December to 30 April) and summer (1 May to 30 November). In all, we collected data from 16 different radio-marked lynx individuals, 5 females with kittens during 10 kill rate study periods for a total of 101 days, 6 adult males during 13 periods for a total of 131 days and 6 solitary adult females during 24 periods for a total of 313 days.

The general radio-tracking routine between the intensive kill rate study periods was taking positions twice a month from an airplane. During the kill rate study periods, initially the sampling scheme consisted of at least one radiolocation per hour around the clock for a period of ten days. In order to increase our efficiency to detect kill sites we modified this to a continuous radio-tracking scheme during 4 hour at dusk and 4 hours at dawn. The averaged number of locations for each lynx was 19 per day during the kill rate periods, which is lower than the expected 24 per day. Sometimes when the lynx were travelling fast it was not possible to get accurate locations by triangulations. Furthermore, after the modification the aim was to get accurate locations when the lynx did not move, which resulted in fewer the 24 locations per day. The modification in radio-tracking scheme was done after preliminary analysing the movement pattern of lynx in relation to roe deer kills. This pattern suggested that the lynx mostly kill roe deer during either dusk or dawn and often return to these kills during the following dusk period.

The radiolocations were plotted on a map, and all clusters of radiolocations were visited as soon as the lynx had left the area. The area around each cluster was searched carefully with the help of a dog to find the remains of a possible kill. The sex and age-class of killed roe deer was determined if possible. We used only two age-classes, juvenile (0–1 year old) or adult (more than 1 year old).

### Roe deer density

Roe deer density was estimated using pellet group counts [36]. We assumed a defecation rate of 22 per 24-hour period [32] and an accumulation period of 200 days, i.e., between leaf fall in early October and the survey in late April. We divided the largest study area (8 000 km^2^) into a grid of 8 x 8 km, giving 120 grid cells. Within each grid cell we randomly selected a 1 x 1 km sampling unit. Within this 1 x 1 km unit we placed 40 circular pellet group count plots regularly spaced (50 m interval) along two parallel 1 km long line-transects spaced 600 m apart. The size of these plot circles was 10 m^2^. In the analyses we treated the 1 x 1 km units as an independent sample of roe deer density. The roe deer survey in the entire study area was performed in 1996, 1998 and 2000 (i.e., three different surveys) in late April and early May, just after snowmelt but before the start of the growing season. A roe deer density index (mean number of roe deer pellet groups per 10 m^2^) was calculated for each radio-tracked lynx home range for each year (June 1 to May 31). For 1997 and 1999 when no pellet counts were done, we used the means between 1996 and 1998 and between 1999 and 2000, respectively. We also estimated the roe deer density (n deer per km^2^) for the medium-sized study area (1200 km^2^) containing 31 of the 1 x1 km pellet count units). This sub-area was selected because we had data on total number of lynx in that area. In the summer and winter 1998/1999 there were 8 lynx individuals in this sub-area, 7 of these were radio-tracked and the 8^th^ lynx was identified by snow tracking (Fig. 1).

We used long-term wildlife survey data from Grimsö wildlife research area (130 km^2^) [for details see 27] to evaluate factors affecting roe deer growth rate. Roe deer density in this area has been surveyed by pellet group counts with a little varying design every year from 1977 till present. In this study we have used data from this long term monitoring program for the period 1985–2005. From 1985 to 1998 the roe deer survey was along 12 transect lines each 5 km long and 400 m apart and with a 1 x 10 m polygon plot every 100 m. In total there were about 550 polygons of 10 m^2^ surveyed every year. From 1997 to 2005 sampling design was changed. The research area was divided into 32 grid cells. Within each grid cell, a 1 x 1 km sampling unit was chosen. Along the perimeter of the sampling unit 10 m^2^ circle plots were spaced every 200 m i.e., 20 circle plots per 1 x 1 km sampling unit. In total there were about 600 circle plots of 10 m^2^ surveyed every year. The roe deer survey was performed in late April and early May. The mean roe deer densities from the two different sampling designs were used for 1997 and 1998 when both sampling designs were used. Total number of red fox dens with litters in the research area has been surveyed in May-June every year since 1973. Findings of cub-scats and remains of fresh prey around the den were used to indicate reproduction. Vole density index, measured as number of voles caught per 100 trap-nights, was based on vole snap trapping in May. Twenty sample areas were systematically distributed over the research area with 50 traps in each sample area. The traps were set for three nights. Data on snow depth were obtained from the meteorological station at Ställdalen (39 km NW of Grimsö, Swedish Meteorological and Hydrological Institute, SMHI). Winter harshness was the summed daily snow depth from the first day with snow in autumn to the last day with snow in spring.

### Analysing kill rate

We tested how kill rate was influenced by lynx social status (females with kittens, males and solitary females, respectively), roe deer density in the lynx home range and season (winter [1 December to 30 April] and summer [1 May to 30 November]). The effect of roe deer density was tested for Type I and Type II functional responses [see 19]. We use a Type II functional response; kill rate = (a*P)/(h+P), where P is prey density, a is the asymptotic kill rate (i.e., the kill rate at high prey density) and h is the half-saturation density (i.e., prey density when kill rate is 1/2 a). We also tested a Type I functional response, kill rate = a+b*P, i.e. kill rate increase linearly with prey density. A Type 0 functional response was tested indirectly, because models not including prey density are Type 0 functional responses.

We tested the effect of lynx social status and season both with and without Type I and Type II functional response. The effect of lynx social status and season was tested to influence the asymptotic kill rate for Type II response and was added in the equation as: kill rate = ((a + b_1_*x_1_ + b_2_*x_2_)*P)/(h+P); where b_1_ and b_2_ are coefficients estimating the effect of the covariates x_1_ and x_2_ on the asymptotic kill rate. For Type I response we add lynx social status and season as: kill rate = a + b_1_*x_1_ + b_2_*x_2_ + b_3_*P. All of our radio-marked lynx individuals were used repeatedly during several kill rate study periods. Therefore we included lynx individual as a random factor in the analyses. The analyses were performed using the R 2.13 [37] and the nlme-library (non-linear mixed effect models). Model selection was based on Akaike information criterion (AIC) corrected for small sample sizes (AICc).

We tested for differences in the number of days spent at the roe deer kill among females with kittens, male and solitary females using linear mixed effect models (R 2.13; lme4-library) with lynx individual as a random factor.

Factors affecting growth rate in roe deer was tested using a linear model (lm, R 2.13) and the models were evaluated using AICc. Growth rate was estimated as: N_t+1_ = λ*N_t_−H_t_ = > λ = (N_t+1_ + H_t_) / N_t_ with a logarithmic transformation; r = log(λ) = b_0_ + b_1_*x_1_ + b_2_*x_2_; where b_1_ and b_2_ are coefficients estimating the effect of the covariates x_1_ and x_2_ on roe deer growth rate.

## Results

### Kill rates

We found 66 lynx killed roe deer, 11 mountain hares, 2 capercaillie, 1 black grouse and 1 greylag goose (*Anser anser*) during 545 radio-tracking days. There was a rather large variation in roe deer density index between the different radio-tracked lynx home ranges, ranging from 0.12 to 0.48 roe deer pellet groups per 10 m^2^, which corresponds to approximately 2.7 to 10.8 roe deer per km^2^. Still there was very weak support for effects of roe deer density both in the linear (Type I functional response) and in the non-linear (Type II functional response) analyses (Table 1). This points to a Type 0 functional response. Neither adding season (ΔAICc = 0.69, Table 1), nor roe deer density index in the linear Type I or the non-linear Type II functional responses improved the model (ΔAICc = 1.33 and ΔAICc = 1.41, Table 1). The model reliability was also very weak for the effects of season and roe deer density index (for both Type I and Type II functional response), as zero were included in the 95% confidence limits for the weighted coefficients (Table 2).

**Table 1:** Models evaluating the variation in Eurasian lynx kill rate on roe deer based on Akaike information criterion (corrected for sample size, AICc) and including only models with a ΔAICc < 2, as well as the null model from south-central Sweden 1996–1999.

Model	AICc	ΔAICc	Model weight
Solitary females^a^ (Type 0)	249.67	0	0.28
Solitary females + Season^b^ (Type 0)	250.36	0.69	0.20
Solitary females + Roe deer density index (Type I)	251.00	1.33	0.14
Solitary females + Roe deer density index (Type II)	251.08	1.41	0.14
Solitary females + Males^c^ (Type 0)	251.08	1.41	0.14
Solitary females + Season + Roe deer density index (Type II)	251.66	1.99	0.10
Null (intercept only) (Type 0)	255.50	5.83	-

Three kinds of functional responses were evaluated; Type 0—no functional response, Type 1—linear functional response and Type II—non-linear functional response.

^a^—Solitary females coded as 1, family groups and males coded as 0.

^b^—Summer coded as 0 and winter as 1.

^c^—Males coded as 1, solitary females and family groups coded as 0.

**Table 2:** Factors influencing Eurasian lynx kill rate on roe deer.

Variable	VRI	Weighted coefficient (± weighted SE)	95% CI		
			lower	upper	
Intercept	-	5.11 ± 1.62	1.94		8.29
Solitary females^a^	1.00	-2.89 ± 1.38	-5.59		-0.19
Season^b^	0.30	1.52 ± 1.41	-1.23		4.28
Roe deer density index (Type II)	0.24	0.098 ± 0.141	-0.178		0.374
Roe deer density index (Type I)	0.14	3.94 ± 3.72	-3.36		11.23
Males^c^	0.14	-1.38 ± 1.35	-4.01		1.26

Parameter estimates (means ± SE) and the variable relative importance (VRI) weights are AICc-weighted model averages from south-central Sweden 1996–1999.

^a^—Solitary females coded as 1, family groups and males coded as 0.

^b^—Summer coded as 0 and winter as 1.

^c^—Males coded as 1, solitary females and family groups coded as 0.

**Fig 2:**
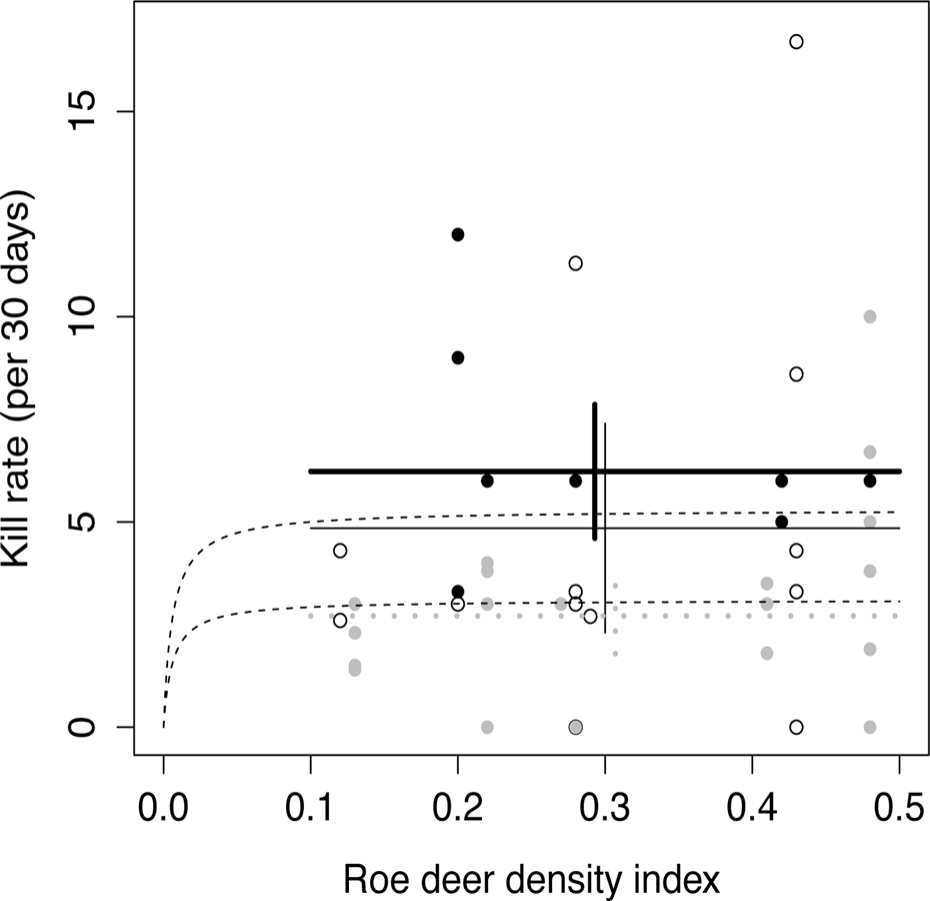
Lynx kill rate (number of killed roe deer per 30 days) in relation to roe deer density index (roe deer pellet groups per 10 m^2^). Females with kittens (black dots and thick line), males (open circles and thin line) and solitary females (grey dots and thick grey line). The horizontal lines indicate mean kill rates for the three categories of lynx, and the vertical lines indicate the 95% confidence intervals of the mean (see Table 3). The thin dotted lines (the upper line females with kittens and the lower line solitary lynx) indicate the Type II functional response from Nilsen et al. [19].

The strongest explanatory power was instead found in “social status” of the lynx. There were large differences in mean kill rates between all three lynx social status with solitary females having the lowest rate (2.7 roe deer/30 days) and female with kittens the highest (6.2 roe deer/30 days; Table 3). The best model included only the effect of lynx social status “solitary female” versus the other two lynx social status (females with kittens and males; Table 1 and Fig. 2), The effect of “solitary female” was also included in all models with a ΔAICc less than 2.

**Table 3:** Eurasian lynx kill rate on roe deer (number of roe deer kill per 30 days) and the number of days on a roe deer kill from south-central Sweden 1996–1999.

Lynx social status	Kill rate(n per 30 days)	Number of days on a roe deer kill
Female with kittens	6.23 ± 0.83 (10)	2.64 ± 0.39 (11)
Males	4.85 ± 1.30 (13)	2.69 ± 0.50 (13)
Solitary females	2.71 ± 0.47 (24)	4.30 ± 0.43 (23)

Mean ± S.E., and sample size within brackets.

The variance in kill rate was significantly higher for males than for females with kittens and solitary females (χ^2^ = 3.84, df = 1, p = 0.05, Bartlett’s test for homogeneity of variances) [38]. Males and females with kittens spent significantly fewer days on a roe deer kill than solitary females (t = 2.30, df = 33, p = 0.03, Table 3). The number of days on a roe deer kill was not significantly affected by season (t = 0.22, df = 32, p = 0.8).

### Effect on the roe deer population

During the summer and winter of 1998/1999 there were three females with kittens (two with one kitten and one with two kittens) and four male lynx radio-marked in the medium-sized study area (1200 km^2^; Fig. 1). From snow tracking we identified one more solitary lynx within an area where we had a radio-marked male but no radio-marked female. We assumed that this unmarked lynx was a female without kitten, because it is typical for lynx social organisation that home ranges of adult individuals of the same sex have little or no overlap [39, 40]. Thus, there were a total of 8 adult lynx in this sub-area. Using the kill rates for the different lynx categories we estimated that the total number of roe deer killed by lynx in this area was 490 (± 70 S.E.) per year. The roe deer density in this sub-area in the winter 1998/1999 was 3.7 roe deer per km^2^ (± 0.91 S.E., n = 31). This gives 4400 (± 1100 S.E.) roe deer within the 1200 km^2^ area that was used by the 8 adult lynx. Thus, the annual predation rate from lynx on roe deer was 11.0% (± 4.2 S.E.).

**Fig 3:**
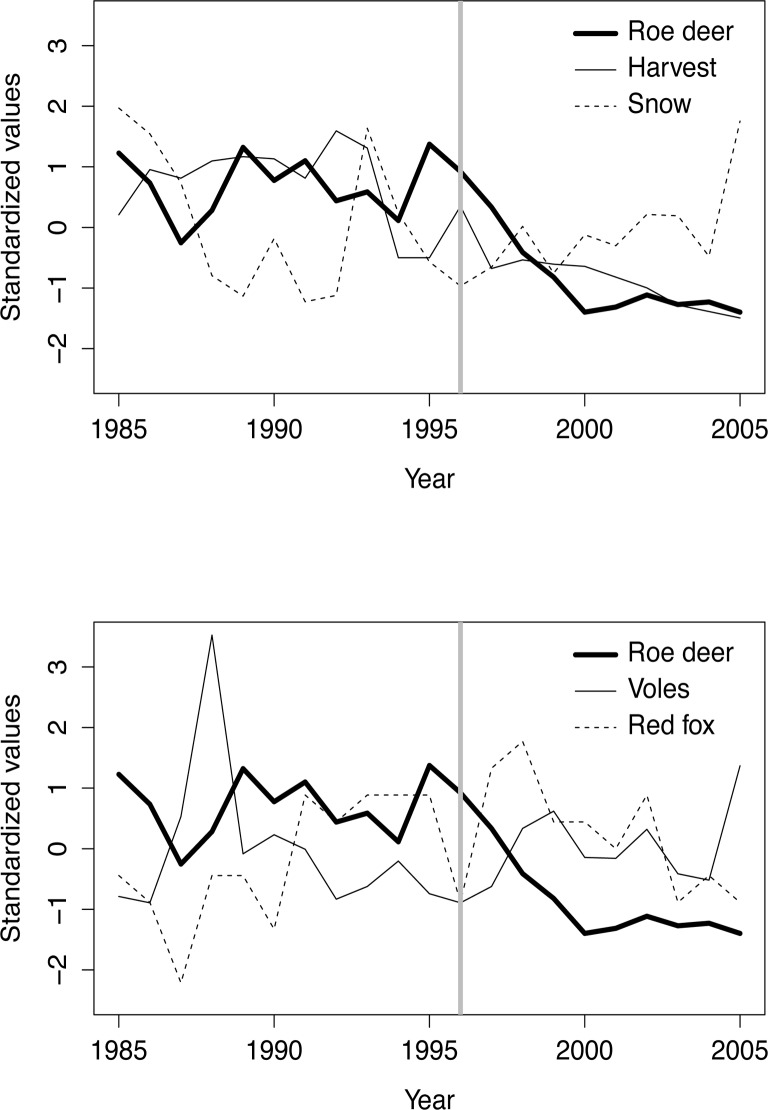
Roe deer density index (mean roe deer pellet groups per 10 m^2^ = 0.239 ± 0.095 st.dev, thick line in both upper and lower part of the graph), roe deer harvest (mean number of roe deer harvested within Grimsö wildlife research area (130 km^2^) = 46.1 ± 28.2 st.dev., thin line upper part), snow depth (mean accumulated snow depth (cm) = 2299 ± 1503 st.dev, dashed line upper part), vole density index (mean number of voles caught per 100 trap nights = 0.596 ± 0.667 st.dev, thin line lower graph), red fox index (mean number of red fox dens with Grimsö Wildlife Research Area with cubs = 6.0 ± 2.26 st.dev, dashed line lower graph). All values are standardized value (x_i_—mean(x) / st.dev(x)). The vertical grey lines indicate the year (1996) of lynx re-colonization of Grimsö wildlife research area.

**Fig 4:**
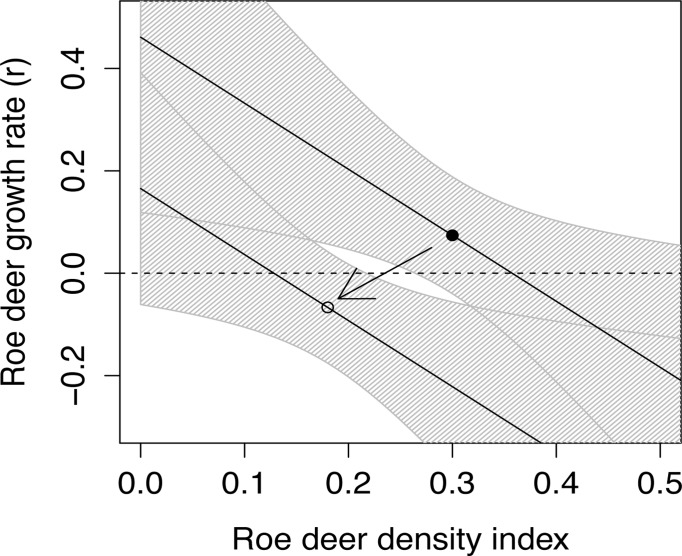
The predicted (± 95% confidence limits) roe deer growth rate (r) in relation to roe deer density index (number roe deer pellet groups per 10 m^2^) and the effect of lynx re-colonization (upper line indicates the before lynx re-colonization (1985–1995) and the lower line after lynx re-colonization (1996–2005)). The black dot shows the mean roe deer density and predicted roe deer growth rate before lynx re-colonization and the open dot shows mean roe deer density and predicted roe deer growth rate after lynx re-colonization.

The annual roe deer growth rate was generally lower after lynx colonized the Grimsö wildlife research area and negatively related to roe deer density index (Figs. 3 and 4). Both these factors were included in three of the four models with a ΔAIC < 2 (Tables 4 and 5). The factors accumulated snow depth and vole density index had less support (Tables 4 and 5). Models including red fox number and roe deer harvest had weaker support than the null model (ΔAICc greater than 3.1).

**Table 4:** Models evaluating the variation in roe deer growth rate within Grimsö Wildlife Research Area 1985–2005, based on Akaike information criterion (corrected for sample size, AICc) and including only models with a ΔAICc < 2, as well as the null model.

Model	AICc	ΔAICc	Model weight
Lynx^a^ + Roe deer density index^b^	-5.05	0	0.39
Lynx + Roe deer density index + Accumulated snow depth	-4.58	0.47	0.31
Vole density index	-3.19	1.86	0.15
Lynx + Roe deer density index + Vole density index	-3.14	1.92	0.15
Null (intercept only)	-2.20	2.85	-

^a^—Lynx coded as 1 after lynx colonization in 1996 and as 0 before 1996.

^b^—Roe deer growth rate = 0.46 (± 0.17 S.E.)- 0.29 (± 0.10 S.E.) * Lynx—1.19 (± 0.54 S.E.) * Roe deer density index.

**Table 5:** Factors influencing roe deer growth rate within Grimsö Wildlife Research Area 1985–2005.

Variable	VRI	Weighted coefficient (± weighted SE)	95% CI	
			lower	upper
Intercept	-	0.42 ± 0.38	-0.32	1.16
Lynx^a^	0.85	-0.31 ± 0.23	-0.75	0.14
Roe deer density index	0.85	-1.34 ± 0.57	-2.46	-0.22
Accumulated snow depth	0.31	-0.000044± 0.000025	-0.000098	0.000010
Vole density index	0.30	0.101 ± 0.083	-0.062	0.265

Parameter estimates (means ± SE) and the variable relative importance (VRI) weights are AIC-weighted model averages.

^a^—lynx coded as 1 after lynx colonization in 1996 and as 0 before 1996.

To estimate the effect of lynx on roe deer growth rate, we have to control for changes in roe deer density between the two periods before and after the lynx re-colonization (Fig. 4). The mean roe deer density index in the period without lynx (1985–1995) was 0.30 pellet groups per 10 m^2^ and the average annual growth rate was r = 0.074 (±0.057 S.E., model uncertainty) or **λ** = 1.079 (± 0.061). In the period after lynx colonized the area (1996–2005), the mean roe deer density index was 0.18 pellet groups per 10 m^2^ and the estimated roe deer growth rate was r = -0.067 (± 0.054 S.E.) or λ = 0.936 (± 0.051). The mean difference in roe deer growth rates between the two periods without and with lynx was Δr = 0.14 (± 0.079 S.E.) or Δλ = 0.14 (± 0.080 S.E.).

## Discussion

The lynx kill rate on roe deer differed among lynx social status; males and females with kittens had a higher kill rate than solitary females (Table 3). This is similar to what Molinari-Jobin et al. [17] and Nilsen et al. [19] found. The reason females with kittens have a higher kill rate than solitary females is most likely caused by a higher food demand. Okarma et al. [41] demonstrated that consumption time was shorter for family groups of lynx than for solitary adult females and solitary subadults of both sexes. They also demonstrated that the consumption was faster, the more kittens that accompanied the female. In our study the data set was too small to differentiate between family groups with different number of kittens.

It is a little harder to understand why male lynx had almost the same kill rate as females with kittens, in spite of their lower nutritional requirement. Okarma et al. [41] also found a similar consumption time in males as in family groups. Lynx males weight about 35% more than females (see below), which corresponds to 22% higher food requirement based on Golley et al. [42] and Munoz-Garcia and Williams [43]. However, this higher food requirement for male lynx is much lower than higher kill rate for males (79% higher) compared with solitary female lynx (Table 3). Instead, we suggest that the reason for the high kill rate in males is social. Males in polygynous species are exposed to a stronger intra-sexual competition than females and spend more time on checking both potential partners and potential competitors, leading to more travelling and larger home ranges [44]. Male lynx have larger home ranges than female lynx [40, 45, 46]. This might also make male lynx more restless than female lynx and thus influence the time they can or want to spend at each kill.

We found very weak support for effect of roe deer density (both Type I and Type II functional response) or of season on lynx kill rate. This is contrary to Nilsen et al. [19] who found a lower kill rate during summer and a Type II functional response of roe deer density on kill rate. However, there are two major differences between the two studies. First, in our study area in Sweden there were only few domestic sheep available during the summer, all sheep are kept on fenced pastures (mostly electric “predator proof” fences), and we did not record any lynx predation on sheep. In the study by Nilsen et al. [19] in Norway, on the other hand, there were large numbers of domestic sheep grazing unattended in the forest during the summer and there was a substantial predation on these by lynx [47]. This additional, easily available, prey type might have made lynx prey less on roe deer during summer in the Norwegian area compared with our study area, where the lynx relied on roe deer alone. Secondly, the roe deer density in our Swedish study area was higher than in the Norwegian area. The roe deer density in our study area was never below 2.7 roe deer per km^2^ while the strong decline in kill rate in the Norwegian study occurred at a density below 1 roe deer per km^2^, and the asymptote for the Type II response in Nilsen et al. [19] was reached already at a roe deer density around 2 roe deer per km^2^, i.e. below our lowest density. Although, we estimated roe deer density using different methods (pellet counts vs. harvest statistics), the lowest density in our study was much higher than the level for the inclination point in the Norwegian study. Therefore, it is unlikely that methodological differences can explain the lack of support for a Type II functional response in our study. The asymptotic kill rate for solitary lynx (both males and solitary females) was between 3.0 and 3.3 roe deer per 30 days in Nilsen et al. [19], compared with 2.7 for solitary females and 4.9 for males in our study. Corresponding values for females with kittens was between 4.7 and 5.8 in the Norwegian study and 6.2 in our study. Thus, the estimated asymptotic kill rates are actually very similar (Fig. 2) and the absence of a Type II response in our study most likely was a consequence of our roe deer density never falling below the breakpoint where the kill rate starts being affected by prey density.

Andersen et al. [48] found no selection of different roe deer categories, i.e., adult males, adult females and juveniles, by lynx. Similar pattern of lynx predation on roe deer was also found by Okarma et al. [41] and Molinari-Jobin et al. [17]. Furthermore, Heurich et al. [23] found that lynx killed prime age roe deer and subsequently the total roe deer mortality increase as lynx recolonized the study area. The mean adult body mass of female lynx was 16.4 kg (± 0.55 S.E., n = 30) and for males 22.1 kg (± 0.51 S.E., n = 21, H. Andrén unpublished data) and for roe deer it was 24.3 kg (±0.19 S.E., n = 147) for females and 25.5 kg (±0.33 S.E., n = 57, P. Kjellander unpublished data) for males. Thus, an adult roe deer weigh only between 10–55% more than an adult lynx. This predator-prey size ratio is similar to mountain lion preying on mule deer and white-tailed deer (*Odocoileus virginianus*) and where there is no selection on age class or condition of the prey [49, 50]. In contrast to this there was a strong selection for fawns and subadults when lynx preyed on red deer (*Cervus elaphus*), which is three times larger than lynx [15, 51]. The non-selective predation put all roe deer in a population at risk for predation, even prime age roe deer with a high reproductive value. This of course increases the potential impact on the prey population. Gervasi et al. [52], using data from a lynx—red fox—roe deer system and from a wolf—brown bear (*Ursus arctos*)—moose (*Alces alces*) system, both in Scandinavia, found that the age composition of the killed prey was a very important factor for determining the impact of predation. They also found that among these four predators, the lynx had the strongest impact on the growth rate of the prey population [52]. Furthermore, Samelius et al. [53] found that roe deer habitat selection was not affected by predation risk, even if 65% of know mortalities in roe deer were due to lynx predation. This indicates an efficient predator irrespective of habitat.

An efficient predator, able to kill prime age prey in good condition, is more likely to make predation additive to other mortality factors [52], which seems to be typical for lynx—roe deer system [23, 24, 54]. This study gives further support for this pattern. Our estimated annual predation rate (0.11 ± 0.042 S.E.) corresponded to the change in growth rate predicted by the models (Δλ = 0.14 ± 0.080 S.E.) including both the effect of lynx re-colonization and roe deer density index (Fig. 4). This suggests that the decline in the roe deer population was mainly caused by predation, and that the predation was mainly additive to other mortality factors in roe deer, although density dependent factors also influence roe deer population dynamics. A review by Melis et al. [18] showed a strong effect of the presence of large predators (lynx and/or wolf) on roe deer density, especially in environments with low plant production. In Norway, roe deer population growth rates were lower in areas with lynx and with harsher climate, suggesting that both lynx and climate have a negative impact on roe deer populations [55].

The population growth rate in the roe deer population within Grimsö wildlife research area was affected by both lynx re-colonization and roe deer density. Thus, both top-down and bottom-up processes influenced the roe deer population. Models with accumulated snow depth and vole density index had only weak support, and we could not detect any effect of red fox number and roe deer harvest. The roe deer harvest has declined dramatically since the lynx arrived, from 0.5 shot roe deer per km^2^ during 1985–1995 to 0.2 during 1996–2005 (harvest statistics from Grimsö Wildlife Research Station). Neither can the decline in the roe deer population be explained by lower roe deer reproduction. The roe deer reproduction (fawns per doe in autumn) within the same study area was related to red fox density and vole density following the alternative prey hypothesis [56, 57, 58], where red fox mainly prey upon voles when they are at high density and switch to roe deer fawns when vole density decreases [28], but voles density index had only a weak effect on roe deer growth rate and there was no effect of red fox density index on roe deer growth rate in this study. Snow depth can affect roe deer growth rate negatively [26], but in this study we could only find a weak support for this. It should be noted though, that we had no real severe snow winter during the study period.

To conclude, our study supports the view of the lynx as a very efficient predator on roe deer capable of reducing roe populations to low levels. Both quantitative estimates of predation rate and behavioural (killing also prime age roe deer) aspects suggest that predation from lynx on roe deer is mainly additive to other mortality factors. Furthermore, this study also suggests that lynx predation acted together with density dependent factors to affect roe deer population dynamics. Thus, in this predator—prey system top-down and bottom-up processes operated at the same time.

## Supporting Information

S1 Dataset Data table for analysing lynx kill rate on roe deer. (TXT)

S2 Dataset Data table for analysing roe deer growth rate. (TXT)

## References

[1] Skogland T. What are the effects of predators on large ungulate populations? Oikos 1991; 61: 401–411.

[2] Errington PL. Predation and vertebrate populations. Quarterly Review of Biology 1946; 21: 144–177.

[3] Bartmann RM, White GC, Carpenter LH. Compensatory mortality in a Colorado mule deer population. Wildlife Monographs 1992; 121: 1–39.

[4] Boyce MS, Sinclair ARE, White GC. Seasonal compensation of predation and harvest. Oikos 1999; 87: 419–326.

[5] Sæther BE. Environmental stochasticity and population dynamics of large herbivores: A search for mechanisms. Trends in Ecology and Evolution 1997; 12: 143–149. 21238011 10.1016/s0169-5347(96)10068-9

[6] Hebblewhite M. Unreliable knowledge about economic impacts of large carnivores on bovine calves. Journal of Wildlife Management 2011; 75: 1724–1730.

[7] Vucetich JA, Smith DW, Stahler DR. Influence of harvest, climate and wolf predation on Yellowstone elk, 1961–2004. Oikos 2005; 111: 259–270.

[8] Tveraa T, Fauchald P, Henaug C, Yoccoz NG. An examination of a compensatory relationship between food limitation and predation in semi-domestic reindeer. Oecologia 2003; 137: 370–376. 12955491 10.1007/s00442-003-1373-6

[9] Griffin KA, Hebblewhite M, Robinson HS, Zager P, Barber-Meyer SM, Christianson D et al Neonatal mortality of elk driven by climate, predator phenology and predator community composition. Journal of Animal Ecology 2011; 80: 1246–1257. 10.1111/j.1365-2656.2011.01856.x 21615401

[10] Bishop CJ, White GC, Freddy DJ, Watkins BE, Stephenson TR. Effect of enhanced nutrition on mule deer population rate of change. Wildlife Monographs 209; 172: 1–28.

[11] Haglund B. Winter habits of the lynx and wolverine as revealed by tracking in snow. Swedish Wildlife Review 1966; 4: 81–299 (in Swedish with English summary). 6013441

[12] Aanes R, Linnell JDC, Perzanowski K, Karlsen J, Odden J. Roe deer as a prey In: Andersen R., Duncan P. and Linnell J.D.C. (editors), The European Roe Deer: The Biology of Success. Scandinavian University Press, Oslo, Norway, 1998 pp. 139–159.

[13] Jedrzejewski W, Schmidt K, Milkowski L, Jedrzejewska B, Okarma H. Foraging by lynx and its role in ungulate mortality: the local (Bialowieza Forest) and the Palaearctic viewpoint. Acta Theriologica 1993; 38: 385–403.

[14] Odden J, Linnell JDC, Andersen R. Diet of Eurasian lynx, *Lynx lynx*, in the boreal forest of southeastern Norway: the relative importance of livestock and hares at low roe deer density. European Journal of Wildlife Research 2006; 52: 237–244.

[15] Gervasi V, Nilsen EB, Odden J, Bouyer Y, Linnell JDC. The spatial-temporal distribution of wild and domestic ungulates modulates lynx kill rates in a multi-use landscape. Journal of Zoology 2014; 292: 175–183.

[16] Jedrzejewska B, Jedrzejewski W, Bunevich AN, Miłkowski L, Krasinski ZA. Factors shaping population densities and increase rates of ungulates in Białowieza Primeval Forest (Poland and Belarus) in the 19th and 20th centuries. Acta Theriologica 1997; 42: 399–451.

[17] Molinari-Jobin A, Molinari P, Breitenmoser-Würsten C, Breitenmoser U. Significance of lynx *Lynx lynx* predation for roe deer and chamois mortality in the Swiss Jura Mountains. Wildlife Biology 2002; 8: 109–116.

[18] Melis C, Jedrzejewska B, Apollonio M, Barton KA, Jedrzejewski W, Linnell JDC et al Predation has a greater impact in less productive environments: Variation in roe deer *Capreolus capreolus* population density across Europe. Global Ecology and Biogeography, 2009; 18: 724–734.

[19] Nilsen EB, Linnell JDC, Odden J, Andersen R. Climate, season, and social status modulate the functional response of an efficient stalking predator: the Eurasian lynx. Journal of Animal Ecology 2009; 78: 741–751. 10.1111/j.1365-2656.2009.01547.x 19486380

[20] Holling CS. The components of predation as revealed by a study of small mammal predation of the European pine sawfly. The Canadian Entomologist 1959; 91: 293–320.

[21] Andersson M, Erlinge S. Influence of predation on rodent populations. Oikos 1977; 29: 591–597.

[22] Abrams PA, Ginzburg LR. The nature of predation: prey dependent, ratio dependent or neither? Trends in Ecology and Evolution 2000; 15: 337–341. 10884706 10.1016/s0169-5347(00)01908-x

[23] Heurich M, Möst L, Schauberger G, Reulen H, Sustr P, Hothorn T. Survival and causes of death of European roe deer before and after Eurasian lynx reintroduction in the Bavarian forest national park. European Journal of Wildlife Research 2012; 58: 567–578.

[24] Melis C, Nilsen EB, Panzacchi M, Linnell JDC, Odden J. Roe deer face competing risks between predators and along a gradient in abundance. Ecosphere 2013; 4: e111 (11 pages).

[25] Kjellander P, Gaillard J-M, Hewison AJM. Density-dependent responses of fawn cohort body mass in two contrasting roe deer populations. Oecologia 2006; 146:521–530. 16341553 10.1007/s00442-005-0188-z

[26] Grøtan V, Sæther B-E, Engen S, Solberg EJ, Linnell JDC, Andersen R et al Climate causes large-scale spatial synchrony in population fluctuations of a temperate herbivore. Ecology 2005; 86: 1472–1482.

[27] Lindström ER, Andrén H, Angelstam P, Cederlund G, Hörnfeldt B, Jäderberg L et al Disease reveals the predator: sarcoptic mange, red fox predation and prey populations. Ecology 1994; 75:1042–1049.

[28] Kjellander P, Nordström J. Cyclic voles, prey switching in red fox, and roe deer dynamics—a test of the alternative prey hypothesis. Oikos 2003; 101: 338–344.

[29] Esseen PA, Ehnström B, Ericson L, Sjöberg K. Boreal forests. Ecological Bulletin 1997; 46:16–47.

[30] Liberg O, Glöersen G. Lynx and wolf survey in Sweden in 2000. Swedish Association for Hunting and Wildlife Management. (in Swedish with English Summary) 2000. 18 pages.

[31] Liberg O. Lodjuret—viltet, ekologin och människan. Svenska Jägareförbundet, Almqvist och Wiksell Tryckeri, Uppsala, Sweden 1997 97 pages (In Swedish)

[32] Cederlund G, Liberg O. Rådjuret—viltet, ekologi och jakten. Svenska Jägareförbundet, Almqvist och Wiksell Tryckeri, Uppsala, Sweden 1995 301 pages. (In Swedish).

[33] Liberg O, Andrén H. The lynx population in Sweden 1994–2004. An evaluation of the census data and methods. Grimsö Wildlife Research Station, Swedish University of Agricultural Sciences Report. ISBN 13: 978–91–976324–0–9. 2006. 60 pages.

[34] Kreeger TJ, Arnemo JM. Handbook of wildlife chemical immobilization. Third edition Sunquest, USA 2007 432 pages.

[35] Arnemo JM, Evans A, Fahlman Å (editors) Biomedical protocols for free-ranging brown bears, wolves, wolverines and lynx. Available: http://www1.nina.no/RovviltPub/pdf/Biomedical%20Protocols%20Carnivores%20March%202012.pdf. Accessed 2015 Feb 11.

[36] Neff DJ. The pellet-group count technique for big game trend, census, and distribution: a review. Journal of Wildlife Management 1968; 32:597–614

[37] R Development Core Team. R: a language and environment for statistical computing. R Foundation for statistical computing, Vienna 2011 10.1016/j.neuroimage.2011.01.013

[38] Sokal RR, Rohlf FJ. Biometry. Third edition Freeman and Company, New York, USA 1995 887 pages.

[39] Breitenmoser-Würsten C, Zimmermann F, Stahl P, Vandel J-M, Molinari-Jobin A, Molinari P et al Spatial and social stability of a Eurasian lynx *Lynx lynx* population: an assessment of 10 years of observation in the Jura Mountains. Wildlife Biology 2007; 13: 365–380.

[40] Mattisson J, Persson J, Andrén H, Segerström P. Temporal and spatial interactions between an obligate predator, the Eurasian lynx (*Lynx lynx*), and a facultative scavenger, the wolverine (*Gulo gulo*). Canadian Journal of Zoology 2011; 89: 79–89.

[41] Okarma H, Jedrzejewski W, Schmidt K, Kowalczyk R, Jedrzejewska B. Predation of Eurasian lynx on roe deer *Capreolus capreolus* and red deer *Cervus elaphus* in Bialowieza Primeval Forest, Poland. Acta Theriologica 1997; 42: 203–224.

[42] Golley FB, Petrides GA, Rauber EL. Jenkins JH . Food intake and assimilation by bobcat under laboratory conditions. Journal of Wildlife Management 1965; 29: 442–447

[43] Munoz-Garcia A, Williams JB. Basal metabolic rate in carnivores is associated with diet after controlling for phylogeny. Physiological Biochemical Zoology 2005; 78: 1039–1056. 16228943 10.1086/432852

[44] Clutton-Brock TH. Review lecture: mammalian mating systems. Proceedings of the Royal Society B, Biological sciences 1989; 236:339–372. 2567517 10.1098/rspb.1989.0027

[45] Linnell JDC, Andersen R, Kvam T, Andrén H, Liberg O, Odden J et al Home range size and choice of management strategy for lynx in Scandinavia. Environmental Management 2001; 27: 869–879. 11393321 10.1007/s002670010195

[46] Herfindal I, Linnell JDC, Odden J, Nilsen EB, Andersen R. Prey density, environmental productivity and home-range size in the Eurasian lynx (*Lynx lynx*). Journal of Zoology 2005; 265: 63–71.

[47] Odden J, Herfindal I, Linnell JDC, Andersen R. Vulnerability of domestic sheep to lynx depredation in relation to roe deer density. Journal of Wildlife Management 2008; 72: 276–282.

[48] Andersen R, Karlsen J, Austmo LB, Odden J, Linnell JDC, Gaillard J-M. Selectivity of Eurasian lynx *Lynx lynx* and recreational hunters for age, sex and body condition in roe dear *Capreolus capreolus* . Wildlife Biology 2007; 13: 467–474.

[49] O’Gara B, Harris RB. Age and condition of deer killed by predators and automobiles. Journal of Wildlife Management 1988; 52: 316–320

[50] Husseman JS, Murray DL, Power G, Mack C, Wenger CR, Quigley H. Assessing differential prey selection patterns between two sympatric large carnivores. Oikos 2003; 101: 591–601.

[51] Okarma H. The physical condition of red deer falling prey to the wolf and lynx and harvested in the Carpathian Mountains. Acta Theriologica 1984; 29: 283–290.

[52] Gervasi V, Nilsen EB, Sand H, Panzacchi M, Rauset GR, Pedersen HC et al Predicting the potential demographic impact of predators on their prey: a comparative analysis of two carnivore–ungulate systems in Scandinavia. Journal of Animal Ecology 2012; 78: 939–959. 10.1111/j.1365-2656.2011.01928.x PMC3440569 22077484

[53] Samelius G, Andrén H, Kjellander P, Liberg O. Habitat selection and risk of predation: re-colonization by lynx had limited impact on habitat selection by roe deer. PLoS ONE 2012; 8(9): e75469 (8 pages) 10.1371/journal.pone.0075469 PMC3777928 24069419

[54] Nilsen EB, Gaillard J-M, Andersen R, Odden J, Delorme D, van Laere G et al A slow life in hell or a fast life in heaven: demographic analyses of contrasting roe deer populations. Journal of Animal Ecology 209; 78: 585–594. 10.1111/j.1365-2656.2009.01523.x 19379139

[55] Melis C, Basille M, Herfindal I, Linnell JDC, Odden J, Gaillard J-M et al Roe deer population growth and lynx predation along a gradient of environmental productivity and climate in Norway. Ecoscience 2010; 17: 166–174.

[56] Hagen Y. Rovfuglene och viltpleien. Gyldendal Norsk forlag (in Norwegian). 1952.

[57] Lack D. The natural regulation of animal numbers. Oxford Univ. Press 1954.

[58] Angelstam P, Lindström E, Widén P. Role of predation in short-term population fluctuations of some birds and mammals in Fennoscandia. Oecologia 1984; 62: 199–208. 28310714 10.1007/BF00379014

